# One hundred years of *Drosophila* cancer research: no longer in solitude

**DOI:** 10.1242/dmm.039032

**Published:** 2019-04-01

**Authors:** Santiago Nahuel Villegas

**Affiliations:** Instituto de Neurociencias, Consejo Superior de Investigaciones Científicas (CSIC) and Universidad Miguel Hernandez (UMH), Campus de Sant Joan, Apartado 18, 03550 Sant Joan, Alicante, Spain

**Keywords:** Cancer, *Drosophila*, Mary Stark, Metastasis

## Abstract

When Mary Stark first described the presence of tumours in the fruit fly *Drosophila melanogaster* in 1918, would she ever have imagined that flies would become an invaluable organism for modelling and understanding oncogenesis? And if so, would she have expected it to take 100 years for this model to be fully accredited? This Special Article summarises the efforts and achievements of Drosophilists to establish the fly as a valid model in cancer research through different scientific periods.

## Abstract (precis)

**Summary:** This Special Article summarises the history of *Drosophila* as a model to understand the cancer problem, from the initial work carried out by Mary Stark 100 years ago to today.

## Origins

At the beginning of the 20th century, the entomologist Charles W. Woodworth projected the use of *Drosophila melanogaster* as a genetic model organism (Sturtevant, 1959). Some years later, Thomas Hunt Morgan isolated a fly strain bearing a mutation that changed the eye colour from red to white; in doing so, he established the link between genes, chromosomes and phenotypes (Morgan, 1910). From there, the concept of gene inheritance started to materialise by the contributions of Morgan's most renowned students, all well accredited in science history. Alfred Henry Sturtevant suggested that genes must be arranged in a linear order and built the first genetic map (Morgan et al., 1920; Sturtevant, 1913), Calvin Bridges established that chromosomes must be the carriers of genes (Bridges, 1916b), and Hermann Joseph Muller demonstrated the association between gene mutation rate and X-ray exposure (Muller, 1928). But, in the shadows of these prominent men, a woman was using flies to address a different question: do chromosomes carry the cause of cancer? She was a member of Morgan's famous Fly Room and the only woman that moved with him from Columbia to Caltech in 1928. Her name, Mary Bertha Stark, may have been forgotten, but her legacy is not.

## Context

In the words of Charles Mayo, one of the most influential cancer experts at the time, ‘cancer continues to be one of the greatest of modern scourges’ (Mayo, 1918), a view perfectly applicable today. Cancer cells were described as lawless entities without self-control, and it was already clear to early oncologists that the single cells travelling through the lymphatic system or into the circulation caused metastasis.

Theories abounded about the causes of cancer. Some of them certainly bold, such as Carpenter MacCarty's proposal that ‘waiting’ or ‘immature’ cells in adult organisms are at the origin of cancer (MacCarty, 1918), a concept intimately linked to cancer stem cells. This idea led Mayo to suggest that cancer can originate from irritation or trauma that demands continued cell repair (Mayo, 1918).

At the time, researchers had recently rediscovered Mendelian laws, and the part chromosomes played in inheritance was a matter of discussion in academic circles. The role of chromosomes in tumourigenesis was speculated about very early on by David Hansemann (Hansemann, 1890), but it was Theodor Boveri who strengthened this idea. From his observation that a balanced number and structure of chromosomes is essential for the normal development of organisms (Boveri, 1902), he hypothesised that the origin of cancer could be a consequence of a chromosome imbalance that causes the cells to divide uncontrollably, thus linking the origin of cancer cells to a genetic abnormality (Boveri, 1914). These observations were also supported by Walter Sutton's studies in the USA. Boveri studied mitosis in sea urchins and *Ascaris* eggs, and cleverly extrapolated his observations to infer the genetic basis of malignancy; yet he seldom studied cancerous tissues. These ideas were highly speculative, and the experimental demonstration of the theory of heredity was provided by Morgan's studies in *Drosophila* (Morgan et al., 1915), while Stark's work provided the experimental support for the theory of cancer as a disease of the chromosomes (Stark, 1918). Fatefully, Stark's description of fly tumours did not show an abnormal distribution of chromosomes as Boveri's hypothesis predicted. Instead, she observed that ‘the growth in question is caused by a sex-linked Mendelian gene that is inherited strictly’, leading Morgan and Bridges to reinterpret Boveri's view and to propose that the cause of cancer may be found in ‘a recurrent somatic mutation of some gene’, unleashing the idea that cancer could be a result of somatic mosaicism (Morgan and Bridges, 1919).

## First wave: dark bodies

Mary Stark based her studies on the original observation by Bridges of the lethal(1)7 strain, the larvae of which developed intense black spots in their body and died at pre-adult stages (Bridges, 1916a). Stark identified these dark bodies as ‘cellular growths somewhat resembling the tumors of vertebrates’ (Stark, 1918). In this pioneering work, Stark presented an exhaustive description of the tumours in larvae, analysing their size, number and timing of appearance. She tried to prolong animal survival by surgically removing the black masses, and by exposing them to X-rays. She also performed tumour transfers to healthy larvae, using small needles, to examine whether the cancer cells can spread and cause host death. These experiments were inconclusive, owing to the high lethality of the surgery itself (she used small pieces of charcoal as a control), but they represent the first attempt at tumour transplantation in *Drosophila*. In complementary experiments, Stark dissolved the tumours and injected the suspension into healthy animals. She identified that the cells in the tumour suspension were responsible for the death of the fly, as flies that received the control solution survived.

A year later, Stark continued describing flies with cancer, now expanding these studies to non-lethal (benign) tumours (Stark, 1919a) and exploring whether *Drosophila* has bona fide metastases, presenting shreds of evidence that both true and ‘artificial’ metastases may co-exist (Stark, 1919b). Based on her observations that the smallest tumours are often lodged within the dorsal aorta, she speculated that ‘cells from the primary tumor have been carried by the blood into the dorsal aorta, where they develop into secondary tumors or metastases’. However, she also observed that large and irregularly shaped primary tumours could be broken and separated into small pieces by pressing them in the body cavity. Once separated, these masses would keep growing, thus producing artificial metastases (Stark, 1919b).

Only a handful of articles investigating tumours in flies were published over the next 50 years, including a few follow-up studies by Stark (Stark, 1935, 1937; Stark and Bridges, 1926). However, a notorious exception derived from the interest of Fernandus Payne, one of the first Drosophilists and Morgan's close collaborator. He had been observing a similar phenotype for years in some fly strains, but never put a name to it until Stark's compelling articles. He handed these flies, which exhibited black masses, to Ira T. Wilson for further investigation, who thus described the existence of other tumour-bearing fly lines (Wilson, 1924). Importantly, using classical genetics, Wilson found that at least three factors (now referred to as genes) need to be present in the same fly to generate a tumour, providing early evidence of oncogene cooperation. The description of new hereditary tumours in *Drosophila* made it clear that flies can develop cancer and that it was not an isolated observation made by Stark.

During the 1940s and 1950s, a few articles aimed to understand the cancer problem using flies (for example, Ardashnikov, 1941; Demerec, 1947a,b; Fabian and Matoltsy, 1946; Gardner and Woolf, 1949; Hartung, 1948; Russell, 1942). Of particular interest is the work of Elisabeth Russell, who expanded the view on the origin of these tumours by suggesting that environmental cues, and not just genetic features, are involved (Russell, 1940). This concept was supported by studies addressing the effect of population density (Hammond, 1938, 1939), temperature shifts (Hartung, 1947) and diet (Friedman et al., 1951) on tumour penetrance. Berta Scharrer and Margaret Lochhead thoroughly reviewed the insights on cancer provided by studies in insects, emphasizing that they should be used as an alternative approach to the study of tumourigenesis. In the same article, the authors exposed their frustration as studies using invertebrates tended to be naively neglected by the scientific community (Scharrer and Lochhead, 1950).

## Second wave: tumour suppressors

In the 1950s, Elizabeth Gateff saw her purpose of following an academic career vanishing after she was declared an enemy of Bulgaria and banned from pursuing higher education, which she later obtained in Germany. Next, she moved to the USA where she joined Howard Schneiderman's group to pursue a PhD studying development and genetics using *Drosophila*, and became a legend by discovering the first tumour suppressor gene.

Like Stark 50 years earlier, Gateff started working with a mutation isolated by Bridges: the *Iethal(2) giant larvae* [*l(2)gl or lgl*], a gene mapped in 1944 (Bridges and Brehme, 1944) and cloned in 1985 (Mechler et al., 1985). In a series of studies, mostly with Schneiderman, Gateff described that *lgl* mutations result in tumours with a genuine malignant phenotype (Gateff and Schneiderman, 1967, 1969, 1974). They found that *lgl* mutant larvae developed malignant tumours in the brain and in the epithelia of the imaginal discs, which were invasive and lethal, but only in homozygous mutant larvae; thus, *lgl* behaved as a tumour suppressor gene. Gateff perfected a serial *in vivo* transplantation technique in adult flies developed by Ernst Hadorn (Hadorn, 1966), and employed it to demonstrate that cells from *lgl* tumours can be transferred from one animal to another an indefinite number of times, resulting in metastasis (Gateff and Schneiderman, 1967, 1969, 1974). This technique has been recently revived (Caussinus and Gonzalez, 2005; Pagliarini and Xu, 2003; Rossi and Gonzalez, 2015) and is becoming a standard method by which to analyse metastatic potential in adult flies.

Back in Germany, Gateff continued describing new tumour suppressors in flies (Gateff, 1982). She was an enthusiastic ambassador of fly models for cancer research for both genetic and epigenetic studies (Gateff, 1978a), at a time when epigenetics was an embryonic concept. In 1978, Gateff wrote an influential article on the merits of using *Drosophila* for cancer studies (Gateff, 1978b), probably inspiring new generations of Drosophilists. Her work propelled a new series of studies in flies, and, although the wave was not overtly surfed, these waters started being navigated. It coincided with a period of significant advances in *Drosophila* investigations that, while having no explicit intentions to translate the results to biomedicine, provided key insights into the role of genes in tumourigenesis. These stunning times, when science was mainly curiosity driven, instead of tilted towards applicability, produced crucial knowledge owing to the use of model organisms that later proved to be crucial to understanding several human diseases (Duronio et al., 2017).

Outstanding work on developmental compartments (García-Bellido et al., 1973) and cell competition (Morata and Ripoll, 1975), a phenomenon that occurs when cells that are less fit than their neighbours are eliminated via short-range cell–cell interaction, made remarkable contributions to cancer research by providing essential information on the mechanisms of growth control and the genes involved. These findings also opened up the possibilities of clonal analysis as a crucial discovery tool (Crick and Lawrence, 1975). For example, it was later demonstrated that cancer cells overexpressing Myc fuel tumour growth by eliminating the surrounding healthy cells (de la Cova et al., 2004; Moreno and Basler, 2004), whereas Myc mutant cells (Johnston et al., 1999) or cancer cells bearing mutations in polarity genes are outcompeted by their wild-type neighbours, resulting in tumour suppression (Brumby and Richardson, 2003). Groundbreaking studies on genes controlling the body plan (Lewis, 1978; Nüsslein-Volhard and Wieschaus, 1980), together with the development of sophisticated genetic tools exclusive to flies (Rubin and Spradling, 1982; Spradling and Rubin, 1982), led to a period throughout the 1980s and 1990s when *Drosophila* dominated the field of developmental biology. The synergy between molecular cloning and entirely novel tools, such as the UAS/Gal4 (Brand and Perrimon, 1993) and FLP-FRT (Golic and Lindquist, 1989; Xu and Rubin, 1993) systems, enabled the engineering of cancer tissues formed by wild-type and oncogenic mutant clones. This new ‘fly power’ enabled researchers to weigh the consequences of gene manipulation, and led to crucial discoveries in developmental signalling cascades that backed the understanding of the biology behind tumourigenesis. For instance, generation of genetic mosaics using the FLP-FRT system led to the discovery and characterization of key components of the Hippo pathway (Justice et al., 1995; Xu et al., 1995), which later proved to be of utmost relevance in cancer (Harvey and Tapon, 2007).

Our knowledge of tumour suppressor genes was further expanded by large-scale mutagenesis screenings involving the mobilization of P elements (Torok et al., 1993; Watson et al., 1991), and, by 1994, at least 50 tumour suppressor genes had been identified in flies (Watson et al., 1994).

Near the end of the 20th century, the fact that flies could develop tumours displaying the full range of human cancer features was accredited (St John and Xu, 1997). The knowledge derived from basic *Drosophila* research was, little by little, conveying invaluable information about the genes and proteins relevant to human cancers. Research on the cell cycle (Edgar and Lehner, 1996; Milán et al., 1996), cell death (Karim and Rubin, 1998; Milán et al., 1997) and epithelial cell–cell interactions (Bilder et al., 2000), together with in-depth studies on the molecular mechanisms of specific tumour suppressors (Ohshiro et al., 2000; Peng et al., 2000), provided a more complete understanding of the different aspects of tumour formation. The last (and definitive?) wave was ready and waiting.

## Third wave: oncogenic mechanisms, drug screens and avatars

The decodification of the fly and human genomes (Adams et al., 2000; Lander et al., 2001) exposed, beyond expectations, an astounding evolutionary conservation of most cellular pathways implicated in development and tumourigenesis.

As the new century dawned, the first report of a fly genetic model of tumour invasion and metastasis (Pagliarini and Xu, 2003), followed by seminal work – now with a clear intention of using *Drosophila* as a model organism for cancer research – firmly positioned flies on the map of cancer models. Consequently, these studies made singular advances in the understanding of tumourigenesis, such as the identification of the part played by cell polarity deficiencies (Brumby and Richardson, 2003; Grifoni et al., 2004; Igaki et al., 2006), oncogenic cell signalling (Read et al., 2004), the role of neural stem cells and asymmetric cell division in brain tumours (Caussinus and Gonzalez, 2005), the non-cell-autonomous tissue overgrowth driven by dysfunction in endocytic components (Moberg et al., 2005; Vaccari and Bilder, 2005) and tumour growth regulation by epigenetic silencing (Ferres-Marco et al., 2006). More recently, many more cancer mechanisms have been identified with work in flies, such as the role of stress signalling in cooperative oncogenesis (Wu et al., 2010), the pro-tumorigenic action of chromosomal instability (Dekanty et al., 2012), mitochondrial dysfunction (Ohsawa et al., 2012), cytokinesis failure and tetraploidy in epithelial tissues (Eichenlaub et al., 2016), and the identification of tumour-expressed systemic hormones involved in cancer-associated cachexia (Figueroa-Clarevega and Bilder, 2015; Kwon et al., 2015). The demonstration that drugs can efficiently block a tumour phenotype in flies (Vidal et al., 2005) opened the gate to *in vivo* screening platforms for anti-cancer drug discovery (Gladstone and Su, 2011; Gonzalez, 2013). The advent of genome-wide UAS-RNAi libraries and the expansion of the fly genetic toolkit boosted research into specific oncogenic mechanisms. As a foremost example of the power of *Drosophila* in biomedical research, flies are currently being engineered to carry the mutations of specific cancer patients, known as avatar flies, and are used to define specific anti-cancer drug cocktails, in an approach that holds tremendous potential for personalised medicine (Kasai and Cagan, 2010; Sonoshita and Cagan, 2017).

**Fig. 1.**
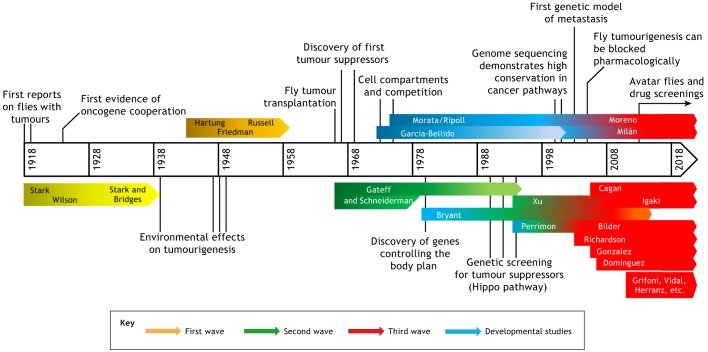
**Timeline showing the key milestones** **and laboratories in the history of *Drosophila* cancer research.** From the initial studies of Mary Stark (first wave), through the breakthrough research of Elizabeth Gateff (second wave) and the developmental studies crucial to understanding tumour biology, and finally to the revival of the fly model for cancer studies at the beginning of the new century (third wave). The scheme includes a few groups that have made key recent contributions to the field (bottom right), as representative of the many laboratories that currently use *Drosophila* to address the cancer problem.

The effort of many scientists established and confirmed the validity of *Drosophila* in cancer research (Fig. 1). This Special Article intends to pay tribute to all of them, and in particular to Mary Stark*.* Her work provided the foundation for using flies as a model to address the cancer problem and has ushered in a century of unparalleled discoveries in the field. We do not know much about her, only that she was there. She resembles the girl from Gabriel Garcia Marquez's *One Hundred Years of Solitude*, who ‘had that rare virtue of never existing completely except for that opportune moment’.
